# Comment on “Should we reframe how we think about physical activity and sedentary behavior measurement? Validity and reliability reconsidered”

**DOI:** 10.1186/s12966-016-0392-8

**Published:** 2016-06-10

**Authors:** Caroline B. Terwee, L. B. Mokkink, L. M. Hidding, T. M. Altenburg, M. N. van Poppel, M. J. M. Chinapaw

**Affiliations:** Department of Epidemiology and Biostatistics and the EMGO Institute for Health and Care Research, VU University Medical Center, F-building MedFac, P.O. box 7057, 1007 MB Amsterdam, The Netherlands; Department of Public and Occupational Health and the EMGO Institute for Health and Care Research, VU University Medical Center, Amsterdam, The Netherlands

**Keywords:** Physical activity, Sedentary behavior, Validity, Reliability

## Abstract

With great interest we read the article by Kelly et al. on the measurement of physical activity (PA) and sedentary behavior (SB) (Kelly P et al. Int J Behav Nutr Phys Act 13:(1) 32, 2016). We appreciate the invitation of the authors to provide feedback on their ideas and we take this opportunity to contribute to the discussion. Our main proposition is that this field can learn much from the field of quality of life research and the methodology developed for validating quality of life questionnaires.

## Main text

With great interest we read the article by Kelly et al. on the measurement of physical activity (PA) and sedentary behavior (SB) [1]. The authors did a great job in presenting the problems and methodological issues associated with assessing reliability and validity of PA and SB instruments. We have encountered many of these issues when performing our systematic reviews on measurement properties of PA questionnaires [2, 3]. We appreciate the invitation of the authors to provide feedback on their ideas and we take this opportunity to contribute to the discussion. Our main proposition is that this field can learn much from the field of quality of life research and the methodology developed for validating quality of life questionnaires.

### The concepts of PA and SB have a lot in common with the concept of quality of life

The concepts of PA and SB and the concept of quality of life are both multi-faceted and rather complex constructs to measure. Both consists of multiple dimensions (e.g. frequency, duration, intensity, and type for PA and physical, mental, and social dimensions for quality of life) and both can be described by multiple domains (e.g. travel, occupational, leisure time, and housework for PA, and symptoms, functioning, and overall quality of life for quality of life) [1, 4].

### The measurement properties relevant for PA and SB instruments are similar to the measurement properties relevant for quality of life (and other) instruments

The relevant measurement properties are the same for all types of instruments. These are reliability (the degree to which the measurement is free from measurement error), validity (the degree to which an instrument measures the construct(s) it intends to measure), and, for instruments used to measure change over time, responsiveness (the ability of an instrument to detect change over time in the construct to be measured) [5]. We agree with Kelly et al. that “terminology is used randomly, synonymously, possibly incorrectly and we all get confused”, especially about different types of reliability and validity. Some examples related to the methodological framework proposed by Kelly et al. will be discussed in the next paragraphs.

Kelly et al. mention the relevance of “behavioural reliability” of PA and SB instruments. They argue that in a reliability study real changes can occur in PA or SB because these behaviors vary from day to day. The same occurs in reliability studies of quality of life instruments. For example, asthma symptoms vary from day to day, which influences the reliability of asthma-specific quality of life questionnaires. Kelly et al. propose to separate the stability of the instrument from the stability of the behavior. In the quality of life field this is not done because reliability is considered to be influenced by various sources of variation, originating from the instrument itself (e.g. poorly formulated questions), the environment (e.g. differences in test circumstances, seasonal influences), or the person itself (e.g. the mood of the person when answering questions). Day to day variation in behavior is considered a source of variation and is therefore included in reliability parameters. Changes between or within persons should exceed these variations in order to conclude that persons are really different or that a person has really changed [6].

We agree with Kelly et al. that measurement error consists of random error and systematic error. All sources of variation can cause random or systematic error. For example, day to day variation can be random (e.g. at one point in time person A may be more physically active than person B and at another point it could be the other way round), or systematic (e.g. when people are more active in summer than in winter). Kelly et al. consider random error an aspect of reliability and systematic error an aspect of validity. In psychometrics, random and systematic error are both considered aspects of reliability. Systematic error is not considered an aspect of validity because at both occasions the same construct is measured. The difference between reliability and measurement error in the COSMIN taxonomy does not refer to a distinction between random and systematic error. The difference is that measurement error is expressed in the units of measurement (e.g. the number of minutes engaged in SB), while reliability expresses the measurement error in relation to the variation in the population (e.g. intraclass correlation coefficient (ICC)) [6].

Kelly et al. propose the concept of ‘context validity’ to assess whether the instrument will give useful information in the proposed context. We doubt the need for an additional aspect of validity because it is well known that all measurement properties are context–dependent [7]. For example, an instrument may be valid for discriminative purposes (e.g. distinguish people with high and low levels of PA) but not for evaluative purposes (e.g. monitor changes in PA over time).

What we miss in the framework by Kelly is the influence of the population. Measurement properties are also population dependent, thus a PA instrument may be reliable for use in an adult population, but unreliable for use in children or the other way round.

We agree with Kelly et al. about their definitions of internal validity (bias) and external validity (generalizability). However, we believe that these terms are redundant in the methodological framework because all measurement properties are aspects of internal validity. External validity is covered by the understanding that all measurement properties are context- and population dependent.

Kelly et al. also propose the concept of ‘proof of concept feasibility’ to pilot test a measure in controlled and free-living settings. We are not in favor of adding a separate term for pilot testing because this is an essential aspect of content validity [8].

Finally, Kelly et al. suggests that the concepts of discriminant validity, divergent validity, and relative reliability and internal consistency needs further discussion. Also here one can learn from the quality of life field. Discriminant validity and divergent validity are aspects of construct validity, similar as convergent validity. Relative reliability parameters express the measurement error in relation to the variation in the population (see above). Internal consistency refers to the interrelatedness of items within an instrument, but is only relevant for instruments that consist of items that are supposed to be highly correlated, which is often not the case with PA or SB instruments [9].

### The standards and criteria used to validate and judge the quality of quality of life instruments can also be applied to PA and SB instruments

If we assume that the relevant measurement properties are the same for all instruments, it is logical that the standards for how the measurement properties should be assessed and the criteria for what constitutes good measurement properties are also similar. For example, there is consensus that test-retest reliability of a questionnaire should be assessed by administering the questionnaire twice to the same group of people, using a time interval in which it is assumed that the people will not change on the construct of interest. The preferred statistical parameters are the ICC (reliability) and the Standard Error of Measurement (SEM) (measurement error). The ICC should be >0.70 and the SEM should be evaluated against what constitutes an important change for patients [10]. If the reliability of a questionnaire is lower than 0.70 because of high day to day variation, we recommend to complete the questionnaire on multiple days and average the scores of the days in order to minimize the measurement error due to day to day variation. This is also done with accelerometer data, where usually the accelerometer is worn on multiple days, and the sum or average value of the measurements of the days is taken.

## Conclusion

The field of PA and SB research can learn much from the field of quality of life research and the methodology developed in this field for validating quality of life questionnaires. We propose to use the COSMIN taxonomy and standards, instead of developing new terms and standards, and build on the experiences of the quality of life field. An explanation and perhaps translation of terminology and some good examples of how these standards can be applied to the validation of PA and SB instruments may be all that is needed. We welcome further discussion on the topic.

## Abbreviations

COSMIN: COnsensus-based Standards for the selection of health Measurement INnstruments
ICC: Intraclass correlation coefficient
PA: Physical activity
SB: Sedentary behavior
SEM: Standard error of measurement

## References

[1] Kelly P, Fitzsimons C, Baker G (2016). Should we reframe how we think about physical activity and sedentary behaviour measurement? Validity and reliability reconsidered. Int J Behav Nutr Phys Act.

[2] Chinapaw MJ, Mokkink LB, van Poppel MN, van Mechelen W, Terwee CB (2010). Physical activity questionnaires for youth: a systematic review of measurement properties. Sports Med.

[3] van Poppel MN, Chinapaw MJ, Mokkink LB, van Mechelen W, Terwee CB (2010). Physical activity questionnaires for adults: a systematic review of measurement properties. Sports Med.

[4] Wilson IB, Cleary PD (1995). Linking clinical variables with health-related quality of life. A conceptual model of patient outcomes. JAMA.

[5] Mokkink LB, Terwee CB, Patrick DL (2010). The COSMIN study reached international consensus on taxonomy, terminology, and definitions of measurement properties for health-related patient-reported outcomes. J Clin Epidemiol.

[6] de Vet HC, Terwee CB, Knol DL, Bouter LM (2006). When to use agreement versus reliability measures. J Clin Epidemiol.

[7] Reeve BB, Wyrwich KW, Wu AW (2013). ISOQOL recommends minimum standards for patient-reported outcome measures used in patient-centered outcomes and comparative effectiveness research. Qual Life Res.

[8] Patrick DL, Burke LB, Gwaltney CJ (2011). Content validity--establishing and reporting the evidence in newly developed patient-reported outcomes (PRO) instruments for medical product evaluation: ISPOR PRO Good Research Practices Task Force report: part 2--assessing respondent understanding. Value Health.

[9] Mokkink LB, Terwee CB, Knol DL (2010). The COSMIN checklist for evaluating the methodological quality of studies on measurement properties: a clarification of its content. BMC Med Res Methodol.

[10] de Vet HC, Terwee CB, Ostelo RW, Beckerman H, Knol DL, Bouter LM (2006). Minimal changes in health status questionnaires: distinction between minimally detectable change and minimally important change. Health Qual Life Outcomes.

